# Predictability of individual circadian phase during daily routine for medical applications of circadian clocks

**DOI:** 10.1172/jci.insight.130423

**Published:** 2019-09-19

**Authors:** Sandra Komarzynski, Matei Bolborea, Qi Huang, Bärbel Finkenstädt, Francis Lévi

**Affiliations:** 1 Medical School, Warwick University, Coventry, United Kingdom.; 2 INSERM-Warwick European Associated Laboratory, INSERM U935, Villejuif, France.; 3 School of Life Sciences and; 4 Department of Statistics, Warwick University, Coventry, United Kingdom.

**Keywords:** Neuroscience, Therapeutics, Clinical practice, Drug therapy, Medical devices

## Abstract

**BACKGROUND:**

Circadian timing of treatments can largely improve tolerability and efficacy in patients. Thus, drug metabolism and cell cycle are controlled by molecular clocks in each cell and coordinated by the core body temperature 24-hour rhythm, which is generated by the hypothalamic pacemaker. Individual circadian phase is currently estimated with questionnaire-based chronotype, center-of-rest time, dim light melatonin onset (DLMO), or timing of core body temperature (CBT) maximum (acrophase) or minimum (bathyphase).

**METHODS:**

We aimed at circadian phase determination and readout during daily routines in volunteers stratified by sex and age. We measured (a) chronotype, (b) every minute (q1min) CBT using 2 electronic pills swallowed 24 hours apart, (c) DLMO through hourly salivary samples from 1800 hours to bedtime, and (d) q1min accelerations and surface temperature at anterior chest level for 7 days, using a teletransmitting sensor. Circadian phases were computed using cosinor and hidden Markov modeling. Multivariate regression identified the combination of biomarkers that best predicted core temperature circadian bathyphase.

**RESULTS:**

Among the 33 participants, individual circadian phases were spread over 5 hours, 10 minutes (DLMO); 7 hours (CBT bathyphase); and 9 hours, 10 minutes (surface temperature acrophase). CBT bathyphase was accurately predicted, i.e., with an error less than 1 hour for 78.8% of the subjects, using a new digital health algorithm (INTime), combining time-invariant sex and chronotype score with computed center-of-rest time and surface temperature bathyphase (adjusted *R*^2^ = 0.637).

**CONCLUSION:**

INTime provided a continuous and reliable circadian phase estimate in real time. This model helps integrate circadian clocks into precision medicine and will enable treatment timing personalization following further validation.

**FUNDING:**

Medical Research Council, United Kingdom; AP-HP Foundation; and INSERM.

## Abstract 2

The prediction of core body temperature bathyphase enables the personalization of treatment timing, thus reducing the risk of adverse events and improving therapeutic outcomes.

## Graphical abstract

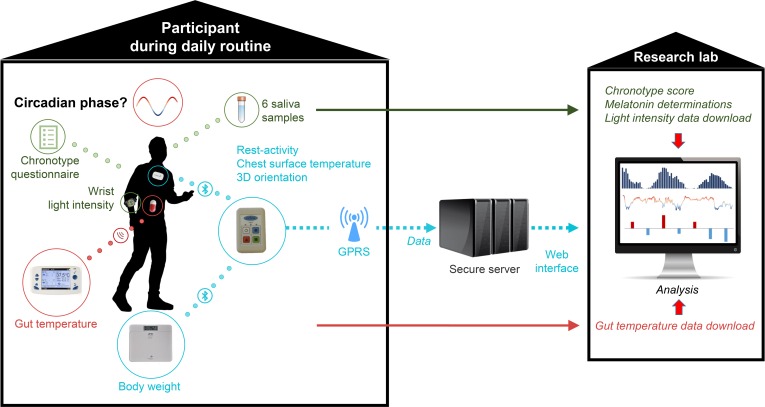

## Introduction

The discovery of the molecular mechanisms of circadian clocks has highlighted a new potential for improving human health through the translation of circadian timing concepts to medical practice (1–3). In mammals, a molecular oscillator involving 15 clock genes generates an oscillation of about 24 hours that rhythmically regulates cellular metabolism, division, and death within each cell (2, 4, 5). The molecular clocks in the cells of all organs are coordinated by an array of physiological rhythms that are generated by the suprachiasmatic nuclei (SCN) in the hypothalamus (6). Thus, the circadian phase of SCN function constitutes a critical measure for inferring timing throughout the organism. The circadian rhythm in core body temperature and that in circulating glucocorticoids, which are controlled by the SCN, play a key role in the coordination of the molecular clocks outside the brain (7–9), while the melatonin secretion rhythm both informs on the circadian phase of the SCN and helps calibrate its period (10). The rest-activity circadian pattern, which integrates lifestyle and social signals, has bidirectional linkage with the SCN and thus can moderate the robustness of SCN rhythmic functions in rodents (11). The circadian timing system (CTS) involves the several components that generate, moderate, or reset the circadian rhythms at cellular, tissue, or whole-body levels, including the retinal light sensor melanopsin, the SCN, the SCN-generated circadian physiology, and the network of molecular clocks in all organs and tissues (2). Studies in healthy humans and in patients with cancer or other diseases have revealed large between-subject differences for the rhythms in rest-activity (12, 13), body temperature (13), circulating cortisol (14), and melatonin levels (15), as well as those in clock gene expression in peripheral tissues (16). More specifically, the extent of the circadian variations (double amplitude of the fitted 24-hour cosine function) in the physiological biomarkers varied by up to several-fold, and the timing of maximum (acrophase) or minimum (bathyphase) varied by up to 12 hours in humans studied during their daily routine (12–16). Occupational schedules can also influence health, through altering CTS function. For instance, night shift work that causes circadian disruption was identified as increasing the risks of breast and possibly other cancers, as well as cardiovascular, gastrointestinal, metabolic, and reproductive disorders (17–19). Recently, circadian rhythm research has reached a critical level where translational applications to human health have become fundamental for many conditions (20, 21). It is clear that treatment timing can largely affect the occurrence of adverse events and efficacy, thus making the consideration of individual differences in CTS function essential for the proper care of patients. Such time dependencies have been demonstrated in randomized clinical trials and meta-analyses involving patients with malignant, cardiovascular, rheumatological, or neurodegenerative diseases (20, 22–25). Similarly, the daily timing of preventive interventions also appeared critical, as shown for vaccination (26). Experimental and clinical studies have further indicated that patients on antibiotics or analgesic medications could benefit from circadian timing optimization of their treatment (2). However, human physiology, experimental chronopharmacology, and clinical chronotherapy trial data have revealed sex-dependent differences in CTS function and stressed their clinical relevance (23, 27). For instance, overall survival was improved significantly in men but not in women receiving the same fixed-time chronomodulated chemotherapy protocol with oxaliplatin and 5-fluorouracil/leucovorin for metastatic colorectal cancer as compared with conventional delivery, as demonstrated in a meta-analysis of 3 international randomized trials involving individual patient data (23).

Thus, it is necessary to reliably determine the circadian phase in real time for each patient, using a noninvasive and simple method, to fulfill the promises resulting from the discovery of circadian clock mechanisms for precision medicine. Novel diagnostic tools have to be developed that aim both at the prevention of circadian disorders, which can lead to chronic diseases or their exacerbation, and at the personalization of clock-based therapies for cancers and other chronic diseases.

Toward this goal, the current study aimed at the continuous and remote determination of the individual subject’s bathyphase (timing of the computed daily nadir) of the overt 24-hour rhythm in core body temperature. New digital health methods and an algorithm were designed here for computing internal circadian phase (INTime) in people whose circadian rhythms were telemonitored in real time during their daily routine.

## Results

### Subjects’ characteristics and chronotype.

**Figure 1:**
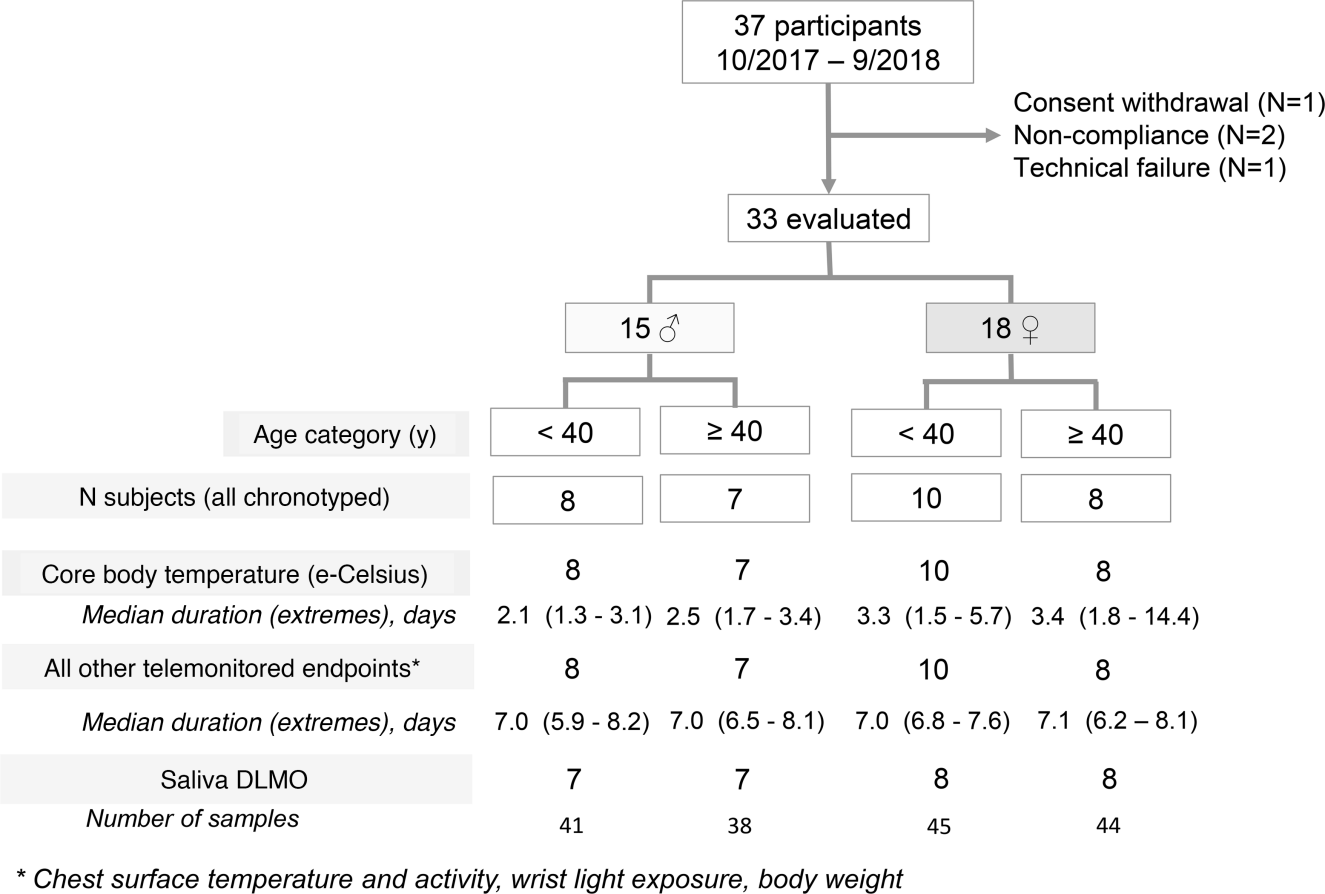
Consort diagram. Flow diagram showing the enrollment of participants, according to sex and age, and the variables that were measured with key features. DLMO, dim light melatonin onset.

Of the 37 recruited participants, 33 provided valid data, including 15 males and 18 females, aged 21–78 years, with a similar distribution according to age (Figure 1 and Supplemental Table 1; supplemental material available online with this article; https://doi.org/10.1172/jci.insight.130423DS1). The majority of participants had no ongoing medical condition and were not taking any medication. Oral contraceptive pills or intrauterine systems were used by 39% of the female participants.

The morningness-eveningness questionnaire scores revealed that chronotype was categorized as “morning” for 15 subjects, “intermediate” for 15 others, and “evening” for 3 participants.

### DLMO.

**Figure 2:**
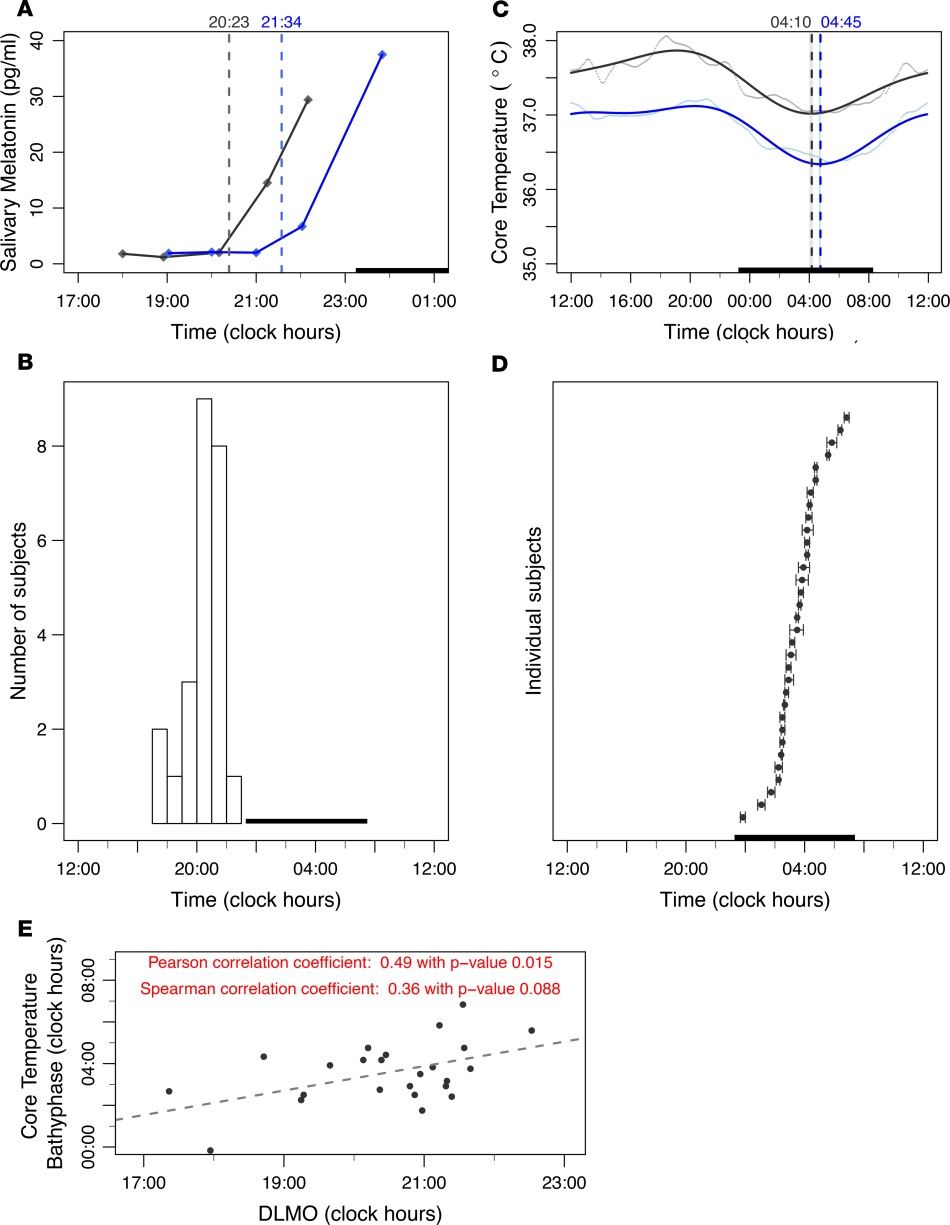
Intersubject variabilities in main circadian biomarkers. (**A**) Salivary melatonin profiles in 2 female participants (28 years old shown in gray and 30 years old shown in blue); the vertical dashed lines represent DLMO, which differed by 1 hour and 11 minutes between both subjects. The dark bar represents the mean sleep spans of both participants. (**B**) DLMO variations among 24 subjects. DLMO could not be computed for 6 participants because of improper or lacking information on sampling times (*n* = 5) or exposure to light greater than 50 lux within 30 minutes of sampling (*n* = 1). The dark bar represents the mean sleep span of the 24 participants. (**C**) Core body temperature patterns in the 2 same participants shown in **A**. Five-minute aggregated data are displayed as dots; the solid curves illustrate the averaged 24-hour profiles according to 2-harmonic cosinor fitting. Bathyphases with 90% CIs estimated by the bootstrap method are indicated with dashed lines and color bands. The dark bar represents the mean sleep span of both participants. (**D**) Core body temperature bathyphase (and 90% CI) variations among the 33 participants. The dark bar represents the mean sleep span of all participants. (**E**) Scatter plots and dashed regression line, with results from both Pearson’s and Spearman’s correlations between DLMO and core body temperature bathyphase for the 24 subjects.

Adequate saliva samples were available for 24 of 30 subjects assessed for this endpoint (80%). Salivary melatonin data were available for computing DLMO using a threshold based on individual baseline values for 12 participants (40%) (Figure 2A). The estimated threshold method based on the pooled 34 baseline values in the same subjects was also applied. Individual DLMOs using both methods differed by –32 minutes to +11 minutes and were strongly correlated (Pearson’s correlation, *r* = 0.96; *P* < 0.001). Thus, we used the estimated threshold method to compute the DLMOs of the 24 subjects. The median clock hour of melatonin secretion onset occurred at 2050 hours with an IQR of 2001 to 2119 hours and individual values spread over 5 hours, 10 minutes (Figure 2B).

### Core body temperature.

Overall, core body temperature time series were provided by both e-Celsius pills in each subject for a median time span of 2.9 days (IQR, 2.0–3.4), ranging from 1.3 up to 14.4 days according to individual gastrointestinal transit. Each of the 66 pills ingested by the 33 participants provided temperature time series over durations ranging from 0.2 to 13.4 days (median, 1.6; IQR, 1.1–2.4). There was an overlap of 24 hours or more for the records by the first and second pill in 13 subjects. Raw temperature data from both pills were correlated within each of these 13 participants, with a median Pearson’s correlation coefficient of 0.86 (IQR, 0.74–0.93) that was highest with a time lag of –2 minutes.

Our cosinor analysis revealed that most individuals displayed a strong 24-hour rhythmic pattern occasionally with an additional prominent 12-hour component (Figure 2C). We found that the precision of the individual bathyphases, as indicated with 90% CIs less than 55 minutes, was much better than that of the corresponding acrophases, whose CIs largely exceeded this value for 6 subjects (18.2%). The median acrophase was located at 1740 hours (IQR, 1520 to 1905 hours), with individual values staggered over 12 hours, 5 minutes. The median bathyphase occurred at 0330 hours (IQR, 0230 to 0415 hours), with individual values spread over a 7-hour span (Figure 2D).

### Rest-activity and surface temperature teletransmitted by chest sensor.

**Figure 3:**
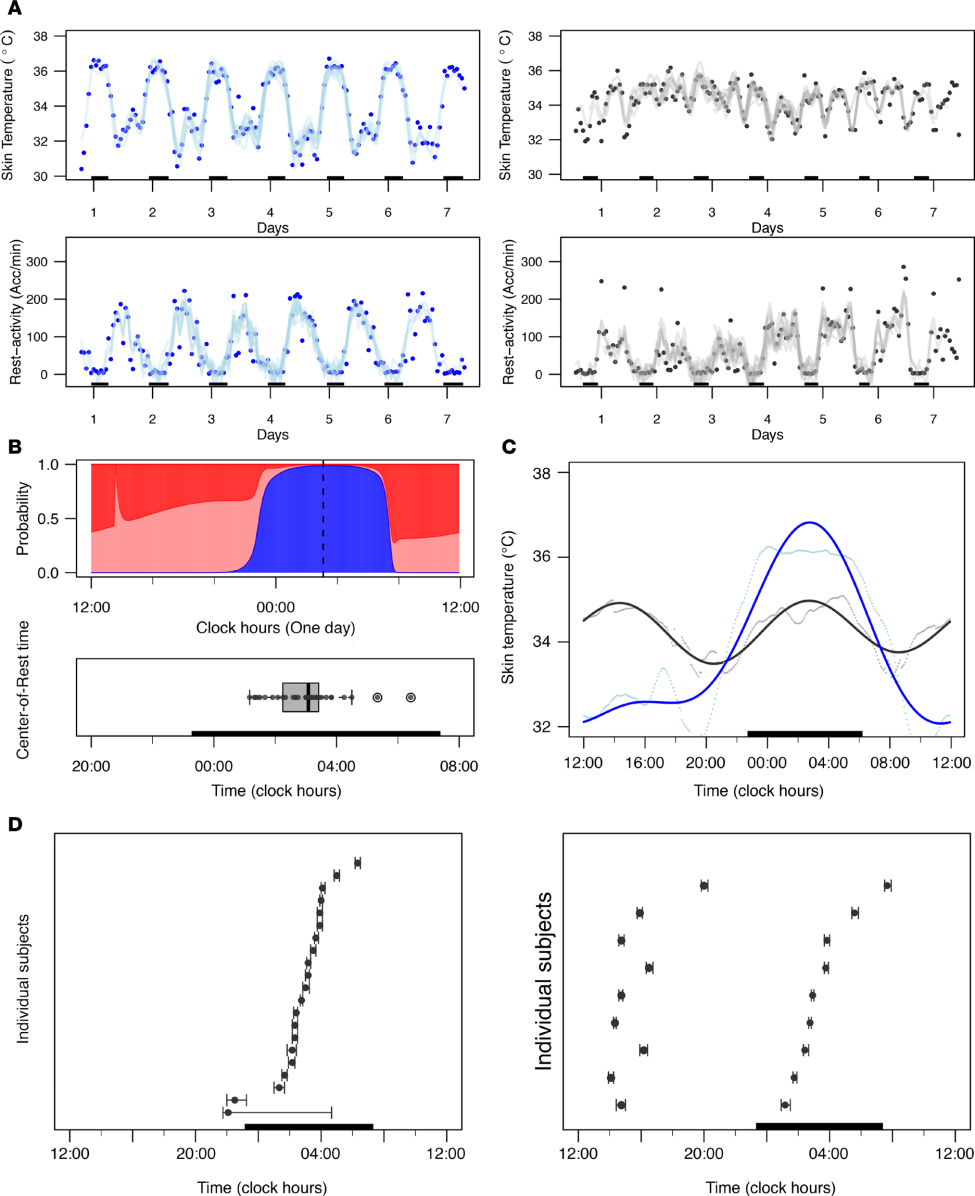
Intersubject variabilities in rest-activity and chest surface temperature. (**A**) Representative examples of chronograms of chest surface temperature (top) and rest-activity (bottom) of 2 participants (blue represents a female, 71 years old; gray, a male, 34 years old). Hourly aggregated data are shown with dots, with solid curves corresponding to Fourier fitting with harmonics estimated using Spectrum Resampling algorithm (28). The dark bars represent the participants’ respective sleeping spans. (**B**) Top: Circadian activity state probability plot from harmonic HMM for a 78-year-old male participant illustrating the computation method of the center-of-rest time. Three activity states were assumed in the HMM, i.e., inactive state (blue), moderately active state (pink), and highly active state (red). The 3 states’ probabilities sum up to 1. The center-of-rest time was computed as the gravity center of the inactive state probability profile (blue), as indicated with a dashed, vertical black line. Bottom: Box plot (5th–95th percentiles) of the center-of-rest times in the 33 participants. The dark bar represents the mean sleep span of all 33 participants. (**C**) Representative examples of the chest surface temperature of the same participants as in **A**. Five-minute aggregated data are shown as dots, and solid curves represent the averaged 24-hour profiles using cosinor fitting. The dark bar represents the mean sleep span of both participants. (**D**) Range of chest surface temperature acrophases (and 90% confidence limits estimated by bootstrap method) of the 24 participants displaying a 24-hour rhythm (left) and the 9 participants with a dominant 12-hour rhythm (right). The dark bar represents the mean sleep span of the corresponding participants.

Rest-activity and temperature time series from the chest surface sensor were available for the 33 participants, for a median duration of 7.0 days (IQR, 6.9–7.3). Large intersubject variations were obvious (Figure 3A). Thus, median number of accelerations per minute ranged from 6 to 135 between subjects, with highest values reaching 331 up to 538. Median chest surface temperature values (5-minute aggregates) varied from 32.6°C to 36.5°C between participants.

Chest surface temperature lowest daily values in fitted curves from individual participants ranged from 32.1°C to 36.4°C and highest daily values from 34.5°C to 36.8°C.

Rest-activity time series displayed regular 24-hour patterns that were highly reproducible from one day to the next in all the subjects, as indicated with prominent 24-hour periods according to spectral analyses (28). Harmonic hidden Markov model (HMM) analyses (29) revealed that the median center-of-rest time was located at 0305 hours (IQR, 0215 to 0325 hours), with individual values ranging over 5 hours, 15 minutes (Figure 3B).

Spectral analyses (13, 28) of chest surface temperature time series identified a dominant 24-hour periodic component for 21 participants (63.6%), and a dominant 12-hour component for 9 of them (27.3%), resulting in 2 daily maxima as shown in cosinor fittings (Figure 3C). No circadian or 12-hour pattern was found for 3 participants (9.1%). According to our cosinor analysis, the median nightly acrophase of chest temperature occurred at 0300 hours (IQR, 0210 to 0355 hours), with individual values spread over 9 hours, 10 minutes (Figure 3D). The corresponding median daily bathyphase that followed the nightly acrophase took place at 1100 hours (IQR, 0940 to 1235 hours), with individual values spread over 10 hours, 25 minutes.

### Noninvasive prediction of core temperature bathyphase.

No statistically significant correlation was found between sex, age, BMI, or concurrent treatment, on the one hand, and the various phase indicators, on the other hand, according to 2-sample *t* test and pairwise Spearman’s correlation (*P* > 0.10). In contrast, circadian phase estimates were correlated to some extent (Supplemental Figure 1). The DLMO was weakly correlated with the core temperature acrophase (Spearman’s correlation, *r* = 0.40; *P* = 0.05) and bathyphase (*r* = 0.36; *P* = 0.09) (Figure 2E). Chronotype score was strongly correlated with center-of-rest time (*r* = –0.70; *P* < 0.001), chest surface temperature acrophase (*r* = –0.60; *P* < 0.001), and core temperature bathyphase (*r* = –0.67; *P* < 0.001). The center-of-rest time was further correlated with both chest surface and core temperature acrophases (*r* = 0.67, and *r* = 0.69, respectively; *P* < 0.001) and core temperature bathyphase (*r* = 0.71; *P* < 0.001). Stepwise model selection identified the “best” regression model for predicting core temperature bathyphase, with an adjusted *R*^2^ of 0.637. The resulting model named “INTime” predicted core temperature bathyphase using the covariates sex (*P* < 0.001) and chronotype score (*P* = 0.009) as well as 2 computed phase indicators from the chest sensor data, namely the center-of-rest time (*P* = 0.033) and chest surface temperature bathyphase (*P* = 0.063) by means of the following estimated equation: core temperature bathyphase = (1.33 × sex) – (0.058 × chronotype score) + (0.472 × center-of-rest time) – (0.145 × chest temperature bathyphase), with sex being coded as 1 for male and 0 for female and phases in hours and decimal hours.

**Figure 4:**
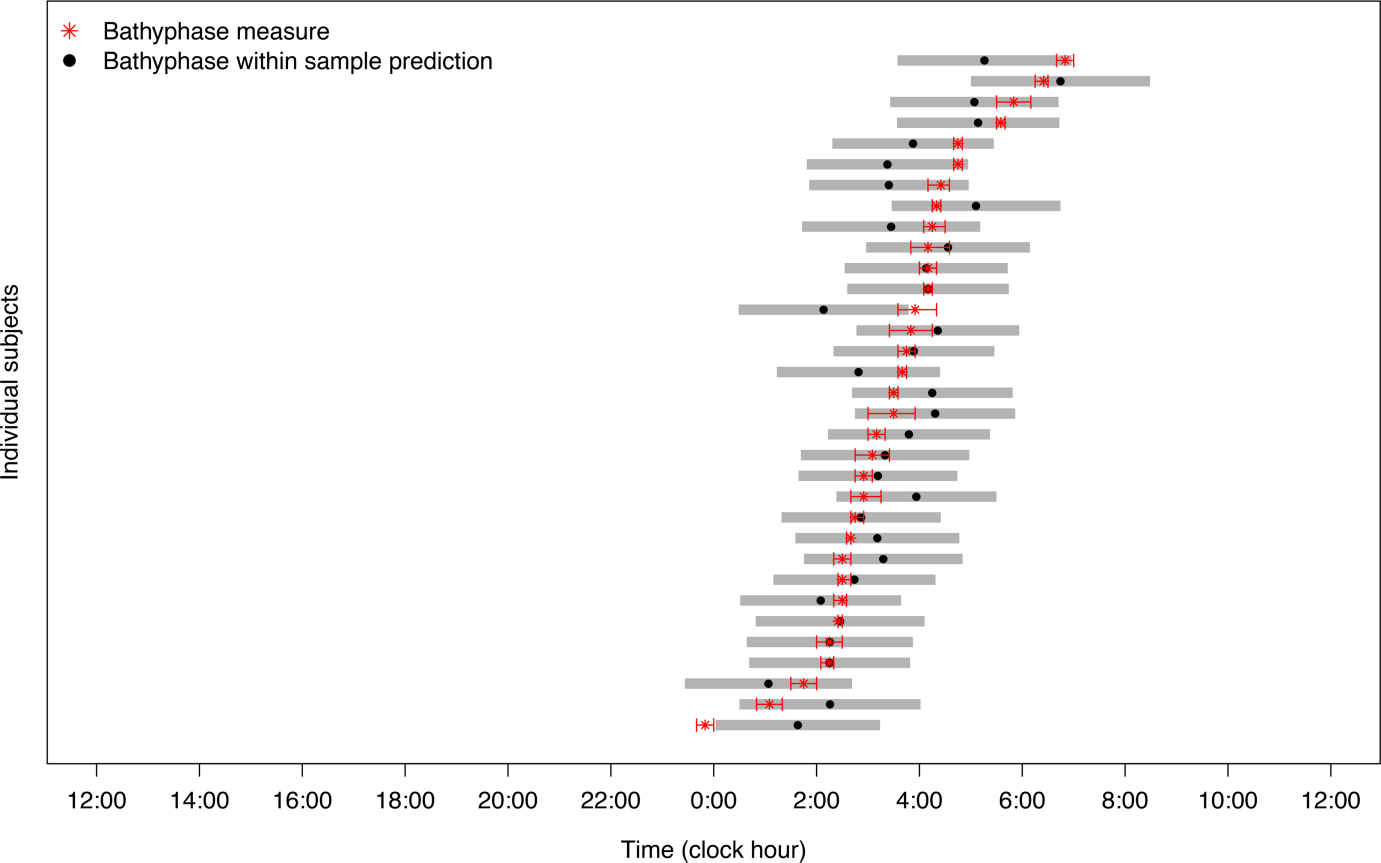
Relations between measured and predicted core body temperature bathyphases in the 33 participants. Red asterisks indicate the computed bathyphases of core body temperature, with their respective 90% confidence limits shown as horizontal limited lines, according to cosinor analysis of real measurements. Dots and gray bands represent the within-sample predicted bathyphases with corresponding 90% prediction bands, as computed using the INTime model.

The accuracy of the predicted core temperature bathyphase (Figure 4) was computed by the distance between the predicted and measured values, whose median was 7 minutes (IQR, –40 minutes to +31 minutes), with individual errors from –106 to +108 minutes. As a result, the fitting error was less than 1 hour for 26 participants (78.8%). In addition, the 90% prediction bands covered most individuals’ measured bathyphase values, i.e., 31 in 33 participants (93.9%), indicating a very satisfactory within-sample prediction accuracy.

## Discussion

Intersubject differences in chronotype make it crucial to perform circadian rhythm measurements without interfering with the daily life of people, to successfully translate and broadly apply circadian clock concepts to precision medicine. The current study represents an important step toward such a goal because it revealed interindividual differences by 7 hours for the bathyphase of core body temperature; by 5 hours, 10 minutes for DLMO; by 5 hours, 15 minutes for center-of-rest time; and by 9 hours, 10 minutes for the acrophase of chest surface temperature, thus highlighting large intersubject variability for these distinct and correlated estimates of circadian phase. The endogenous circadian rhythms in core body temperature, as continuously recorded using a rectal probe (30), and those in circulating melatonin concentrations were robustly coordinated in healthy humans. As a result, the bathyphase of core body temperature has been largely used as an adequate reference for the endogenous circadian phase in humans (31, 32), based on studies performed under constraining constant routine protocols in human chronophysiology laboratories (30, 33). Although salivary DLMO at home might have proved a precise indicator of circadian phase, it could be estimated in only 80% of our very compliant participants. Reasons involved occasional environmental light contamination both outside and at home, possible food contamination by melatonin-containing aliments, and need for alterations in daily and familial routine, including meal timing. To circumvent such drawbacks and to enable clinical applications of circadian clocks, the current study has identified a noninvasive method that provides a precise and continuous estimate of individual circadian bathyphase of core body temperature in real time from remote people during their daily routine. Within-sample accuracy was less than 1 hour for 78.8% of the participants. The use of this model in medical practice requires information on sex, score from the chronotype questionnaire, and 2 circadian timing parameters extracted from chest rest-activity and surface temperature monitoring. Both of them are easily amenable to automatic, real-time computation out of teletransmitted time series being recorded during the daily routine of the person. We expect that INTime will enable circadian timing of treatments, i.e., chronotherapy, to irreversibly complement the basic principle of today’s toxicology, “The dose makes the poison.” This paradigm, which was proposed by Paracelsius more than 500 years ago (2), has driven the current adjustment of drug dose levels to body weight or surface area, pharmacokinetics, or drug polymorphisms, which have become indispensable information for both regulatory approval and safe medical use of medications. Although there is strong evidence that time of day of treatment delivery can matter as much as dose (2, 3, 22–24, 26, 27), we have been lacking metrics for the determination of optimal treatment timing in individual patients. Indeed, results from randomized clinical trials and meta-analyses have shown that the patients’ benefits resulting from drug timing could be as large as 5-fold, yet they could depend upon patients’ sex and CTS function (23, 27). The need for the personalization of treatment timing was further highlighted by up to 8 hours’ difference in optimal timing of the anticancer drug irinotecan, as a function of mouse sex and genetic background. In this large study, optimal timing was predicted by a mathematical model combining the circadian mRNA expression patterns of clock genes Bmal1 and Rev-erbα in the liver or colon, which also governed the key pharmacological mechanisms of this drug (34, 35). Recent results have further highlighted consistent relations between 24-hour temperature cycles and circadian patterns in metallodrug toxicity both in vitro and in vivo (36). The findings are in line with previous studies linking the circadian rhythms in mouse tolerability for 16 anticancer drugs to the intraperitoneal temperature cycle (37).

Both the limited sample size available for DLMO estimations and the weak correlations between DLMO and other timing indicators precluded any attempt toward the search for a prediction model of DLMO. Moreover, the ability to reset most peripheral clocks with physiological temperature cycles but not with melatonin supported a potential key role of this rhythm for the biomedical applications of circadian clocks. The limitations of our study involve the measurement of core body temperature within various segments of the gastrointestinal tract and the lack of a validation sample of the INTime model. Previous studies have shown that the circadian patterns in body temperature measurements were very similar if taken from gut using an ingested pill or from the rectum using a dedicated probe (38), thus supporting gut temperature bathyphase as a reliable circadian phase biomarker. Moreover, INTime predicted circadian phases to range from 0155 to 0705 hours in a distinct cohort of 18 healthy subjects whose sex, chronotype, center-of-rest time, and chest surface temperature bathyphase had been determined previously (13). In this independent sample, median, IQR, and extremes of predicted individual circadian phases matched those in the current study (Supplemental Figure 2) very well, although accuracy could not be computed because core body temperature rhythms were not recorded. By bootstrapping the residuals of the INTime model fit, we obtained pseudo data sets with arbitrary size *n*. By applying 1000 Monte Carlo simulation trials, we found that *n* = 600 and *n* = 1000 samples would be required to stabilize the INTime model fit so that the corresponding 90% and 95% CIs of the adjusted *R*^2^ were less than 10% of the given value of 0.637.

Our findings have a major potential impact for the reduction of severe adverse events from treatments, whose reduction represents a critical challenge for improving patient quality of life, treatment compliance, treatment efficacy, and human health care cost burden. As an example, a 10.8-fold increase in the yearly rate of emergency visits for cancer treatment–related toxicities has been documented over 10 years in a large US study, where 91% of the emergency visits translated into emergency admissions, and 4.9% of deaths, resulting in related costs of billions of US dollars (39).

In conclusion, using a teletransmitting dual-function chest sensor and INTime, treatment timing can be personalized both between and within patients, thus potentially reducing adverse events and improving therapeutic outcomes. Such personalized chronotherapy deserves prospective testing and could help invert the steadily rising economic burden of treatment morbidities in cancer and chronic diseases.

## Methods

### Study design and human subjects

**Figure 5:**
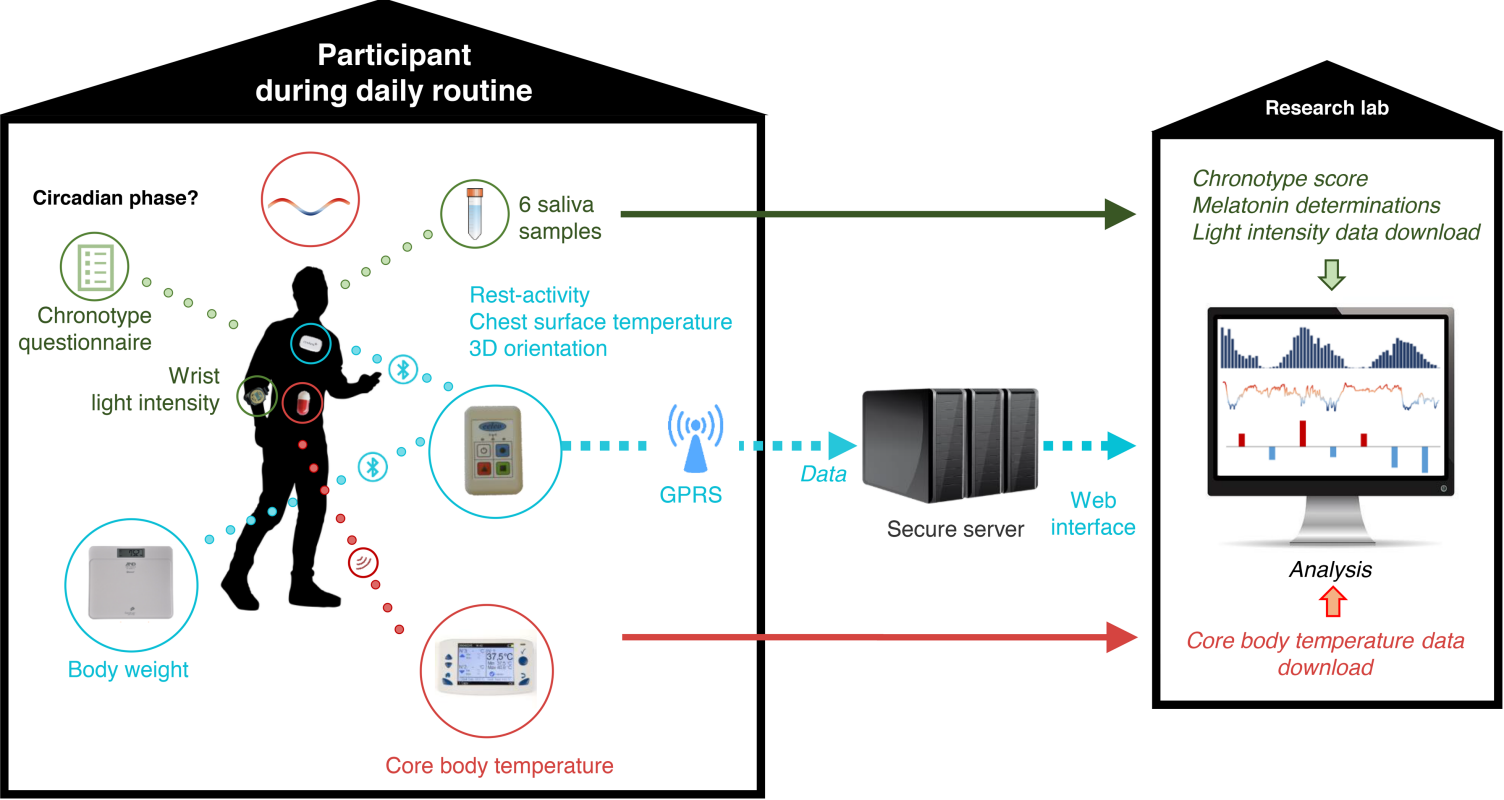
Study picture. Schematic description of the study design. GPRS, General Packet Radio Service. Icon credit: Pixabay (https://pixabay.com/). User license available at https://creativecommons.org/licenses/by/4.0/

The study aimed at (a) the estimation of the internal circadian phase during daily routines, a process that usually requires a constraining circadian physiology protocol in the laboratory (30, 33), and (b) the assessment of the relevance of age and sex to the noninvasive circadian biomarkers selected for informing on the CTS during human daily routines (Figure 5).

We aimed to recruit 30 adult volunteers stratified by sex and age above or below 40 years with valid data.

Eligibility criteria included the ability to work or to perform usual activities and to be aged 18 years or more. Exclusion criteria involved any uncontrolled pathological or psychological condition; any known gastrointestinal disease; any ongoing treatment with glucocorticosteroids, melatonin agonists or antagonists, lithium, or analgesic; any contraindication to the use of electronic devices; and night shift work or crossing of more than 3 time zones within the past 4 weeks. Volunteers were recruited locally through flyers and advertisements in newsletters and local journals. The study participants were asked not to change their free-living daily schedule throughout the study, except on the evening when they were to collect 6 hourly samples at home in dim light starting at 1800 hours until usual bedtime.

### Data collection and processing

Main characteristics of the subjects, including sex, birth date, marital status, professional activity, and past and current illnesses and treatments, were recorded upon study entry. Chest surface temperature, activity, and 3D orientation were recorded every minute for 1 week using the PiCADo mobile eHealth platform (13). These 3 variables were measured using a chest sensor (Movisens) and a pocket-sized gateway (Eeleo). Anonymous data were transmitted via Bluetooth from the sensor to the connected gateway and then transmitted to a secure and dedicated server via GPRS every 24 hours. Because the devices used were not waterproof, their short removal was allowed for showers, baths, or occasional needs. A body weight scale was also connected to the gateway via Bluetooth, with daily measurements acquired before breakfast.

Saliva was self-collected by the participants using saliva collection aids (SalivaBio Passive Drool, Salimetrics) and 2-mL micro tubes (SARSTEDT AG & Co.). Participants were asked to collect 5 hourly samples at home during an evening in dim light starting at 1800 hours and a sixth one at 2300 hours or before retiring, whichever came first. Dim light conditions were verified using a wrist actigraph (Motionlogger Micro Watch, Ambulatory Monitoring Inc.), with all subjects being reminded not to occlude the light sensor with sleeves. A salivary sample was considered invalid for melatonin determination if light intensities greater than 50 lux (40) had been recorded by the wristwatch light detector within the 30 minutes preceding collection.

Core body temperature was measured using an electronic, ingestible pill (e-Celsius Performance pill, BodyCAP Medical). Participants were asked to ingest 1 such pill in the morning for 2 consecutive days. Data were transmitted via radio frequency to a dedicated monitor (e-Viewer Performance Monitor, BodyCAP Medical). Data were transferred from the monitor to the computer of the biomedical engineer after both pills had been eliminated through the stools. The abnormally low or high core body temperature values in the first few hours of recording were deleted because they were typically due to the temperature of food or drink ingestion. Data from the first ingested pill were used until pill elimination in the feces. Data from the second pill were used thereafter to obtain a complete time series. Participants provided a detailed diary with time of awakening and retiring, mealtimes, intense activity times, and medication times (if any).

Chronotypes were determined using the largely validated morningness-eveningness self-assessment questionnaire (41).

### Data management

Teletransmitted chest sensor data were stored on the server based on HL7 standards (international standards for transfer of clinical and administrative data). Data were downloaded from the server to the computer of the biomedical engineer only. Anonymized data were saved on a secure storage server according to the national Data Protection and Freedom of Information Acts guidance. Data transmission was inspected at least twice a week during the monitoring sessions to ensure adequate functioning.

e-Celsius temperature data from the ingestible pills were received on the BodyCAP monitor. Similarly, after retrieval of the monitor from the subjects, data were downloaded and saved on a secure storage server according to the national Data Protection and Freedom of Information Acts guidance.

### Statistics

#### Salivary melatonin secretion.

DLMO is commonly computed as the time when melatonin concentration in plasma or saliva exceeds a threshold computed as the mean of 3 consecutive daytime values before rise plus twice the standard deviation of these 3 points (42). For those subjects with insufficient baseline data, an estimated threshold value was computed as the mean plus twice the standard deviation of the pooled baseline melatonin values in the subjects with adequate baseline data. This estimated threshold was first validated in the subjects with adequate baseline data by Pearson’s correlation, before its use for all the subjects.

#### Chest surface temperature and core body temperature.

A single core temperature time series linked the temperature data measured by the first pill, until its elimination, and the second one, afterward. The temperature data were first aggregated by 5-minute mean and smoothed using a 1-hour moving average window, then computed as an averaged 24-hour profile. We further fitted the following 2-harmonic cosinor model (43), with periods *T*_1_ = 12 hours and *T*_2_ = 24 hours to describe the average day oscillation of both chest surface and core body temperatures based on prior evidence (13):

(Equation 1)
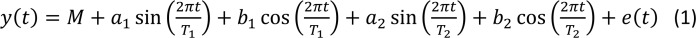


where *y*(*t*) is the temperature at time *t*; *M* is the mesor (mean level of the fitted cosine function); *a_1_* and *a_2_* and *b_1_* and *b_2_* are the coefficients of the cosinor model, and *e*(*t*) is the error. Given the periods *T*_1_ and *T*_2_, the coefficients were estimated by least-squares linear regression. We report the overall acrophase *θ̂ _max_*, i.e., time of maximal value in the fitted values ŷ(*t*), and the overall bathyphase *θ̂ _min_*, i.e., time of minimal value in ŷ(*t*). Note that for core body temperature, we considered mainly bathyphase because it could be identified with a better precision than acrophase in most individuals. Ninety percent CIs for *θ̂ _max_* and *θ̂ _min_* were evaluated and reported by applying *n* = 1000 bootstrap trials (44).

#### Telemetric rest-activity.

A recently developed 24-hour harmonic HMM approach (29) was fitted to the data to compute numerical quantifiers that were associated with circadian rhythm in rest-activity data. The HMM approach categorizes the actigraphy measurement into 3 states, namely inactive/rest, moderately active, and highly active, in a probabilistic way. We focused on the inactive/rest state, in particular the center-of-rest time over a 24-hour span, which provided an estimation of the average center-of-rest time point during the recording period.

#### Prediction of core body temperature bathyphase.

The main aim of the study was to provide a method for the continuous and remote estimation of the individual subject’s bathyphase of the overt 24-hour rhythm in core body temperature. The potential for core temperature bathyphase to be predicted by DLMO, rest-activity, chest surface temperature, and chronotype as well as the subject’s characteristics was investigated using a multivariate linear regression model. Potential predictors were first explored via pairwise Spearman’s correlations. An initial regression model was formulated by considering all phases, with sex, age, and BMI, as explanatory variables. A parsimonious regression model was then identified by a stepwise model selection procedure based on corrected Akaike’s information criterion for small sample size using the *R* function *StepAICc* (45).

Significance of explanatory variables was also tested by 2-tailed *t* test where a possible significant effect was considered for *P* values smaller than 0.1. The distribution of the time distance between predicted and real core body temperature bathyphase measures (residuals) was computed to study the reliability of the prediction. A prediction accuracy of less than 1 hour was considered precise enough for clinical applications.

### Data access statement

R-codes to obtain the datasets corresponding to the salivary melatonin profiles, the core body temperature patterns, and the rest activity and chest surface temperature patterns displayed in Figure 2, A and C; and Figure 3, A and C, as well as the R-codes for cosinor analyses are available at https://github.com/huang1010/JCI-insight

### Study approval

The protocol and subsequent amendments were approved by the Ethical Committee of Warwick University (REGO-2017-2055). The study was conducted according to the Helsinki Declaration (46). The subjects provided signed informed consent documents before their participation in the study.

## Author contributions

The study was co-conceived by SK, MB, and FL. Funding resources were obtained mainly by FL, and the study management was carried out by SK and MB. Technical supervision was performed by SK and MB. Methods were designed by SK, MB, QH, BF, and FL. SK and MB included participants in the study. Data were collected and curated by SK and MB. Statistical methods development and programming were performed by QH. Data analysis was performed by SK and QH. Data interpretation involved SK, MB, QH, BF, and FL. The manuscript drafting and its editing and final approval involved SK, MB, QH, BF, and FL.

## Supplementary Material

Supplemental data

## Version 1 08/20/2019

In-Press Preview

## Version 2 09/19/2019

Electronic publication
