# Supplementary material for: Predictability of individual circadian phase during daily routine for medical applications of circadian clocks
Source: JCI Insight. 2019 Sep 19;4(18):e130423. doi: 10.1172/jci.insight.130423 (PMC6795290; doi:10.1172/jci.insight.130423)
Supplement: Supplemental data [file jciinsight-4-130423-s183.pdf]

**Table S1: Participants' main characteristics**

| | Number of subjects | % |
|---|---|---|
| **Sex** | | |
| M | 15 | 45.5% |
| F | 18 | 54.5% |
| **Age** | | |
| < 40 y.o. | 18 | 54.5% |
| ≥ 40 y.o | 15 | 45.5% |
| **BMI** | | |
| Less than 18.5 - underweight | 0 | 0% |
| 18.5 to 24.9 - healthy weight | 18 | 54.5% |
| 25 to 29.9 - overweight | 13 | 39.4% |
| 30 to 39.9 - obese | 1 | 3.0% |
| 40 and over - morbidly obese | 1 | 3.0% |
| **Work Status** | | |
| Student | 11 | 33.3% |
| Worker | 15 | 45.5% |
| Retired | 7 | 21.2% |
| **Lifestyle** | | |
| Sedentary | 12 | 36.4% |
| Sporty | 4 | 12.1% |
| Variable-Irregular | 17 | 51.5% |
| **Chronotype** | | |
| Definite morning | 5 | 15.2% |
| Moderate morning | 10 | 30.3% |
| Intermediate | 15 | 45.5% |
| Moderate evening | 3 | 9.1% |
| Definite evening | 0 | 0% |
| **Medical condition** | | |
| None | 22 | 66.7% |
| Past | 3 | 9.1% |

| | | |
|---|---|---|
| Ongoing | 8 | 24.2% |
| **Main medical conditions** | | |
| Mild high blood pressure | 3 | 9.1% |
| Hypercholesterolemia | 2 | 6.1% |
| **Medication** | | |
| None | 25 | 75.8% |
| One | 3 | 9.1% |
| > Two | 5 | 15.1% |
| **Most frequent medications** | | |
| Antihypertensive | 4 | 12.1% |
| Metabolic disorder therapy | 3 | 9.1% |
| **Contraceptive methods** | | |
| None | 6 | 18.2% |
| Pill | 5 | 15.1% |
| Intrauterine device or system | 3 | 9.1% |
| Not applicable (menopausal females) | 4 | 12.1% |
| Not applicable (males) | 15 | 45.5% |

## Figure S1: Multiple correlation matrix of phase indicators

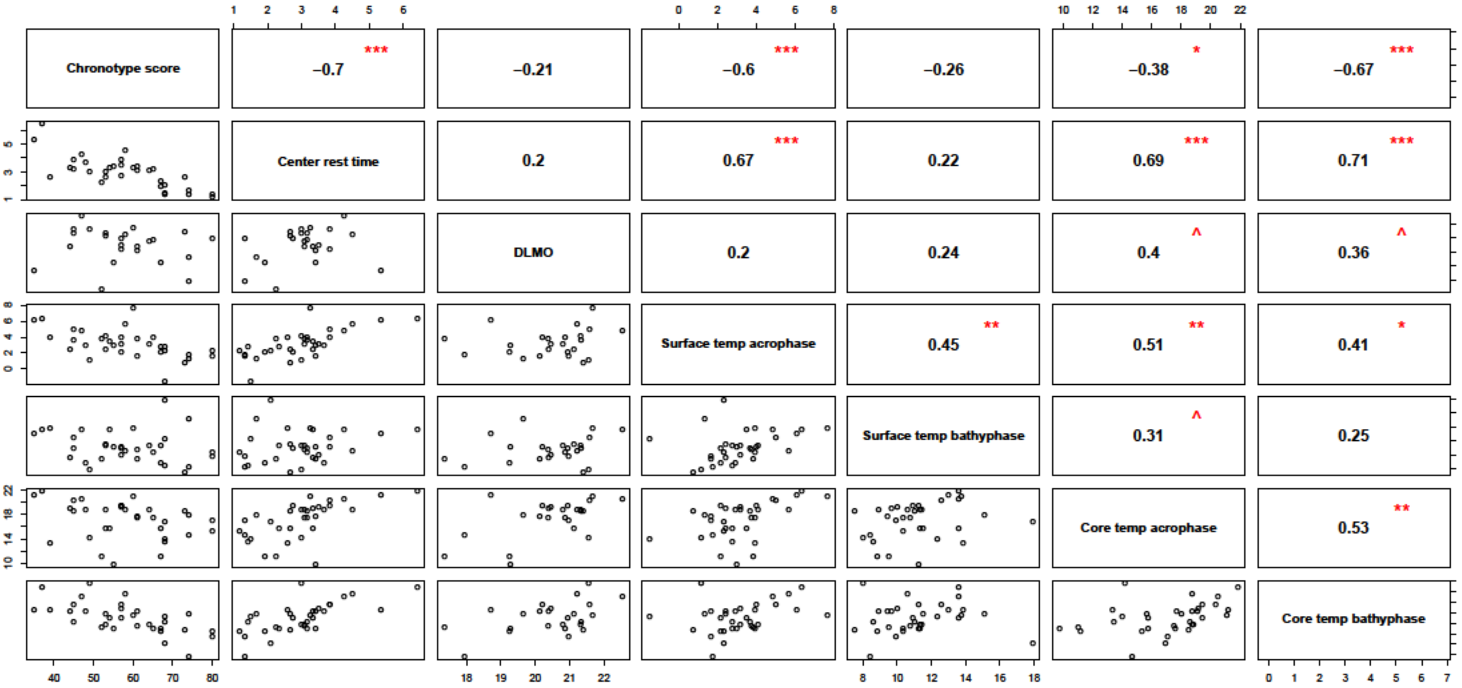


Spearman correlation coefficients with stars indicating p-value levels,

i.e. 0 '***' 0·001 '**' 0·01 '*' 0·05 '^' 0·1.

*DLMO stands for Dim Light Melatonin Onset*

**Figure S2: Comparative distribution of circadian phase estimates in current study and independent data set.**

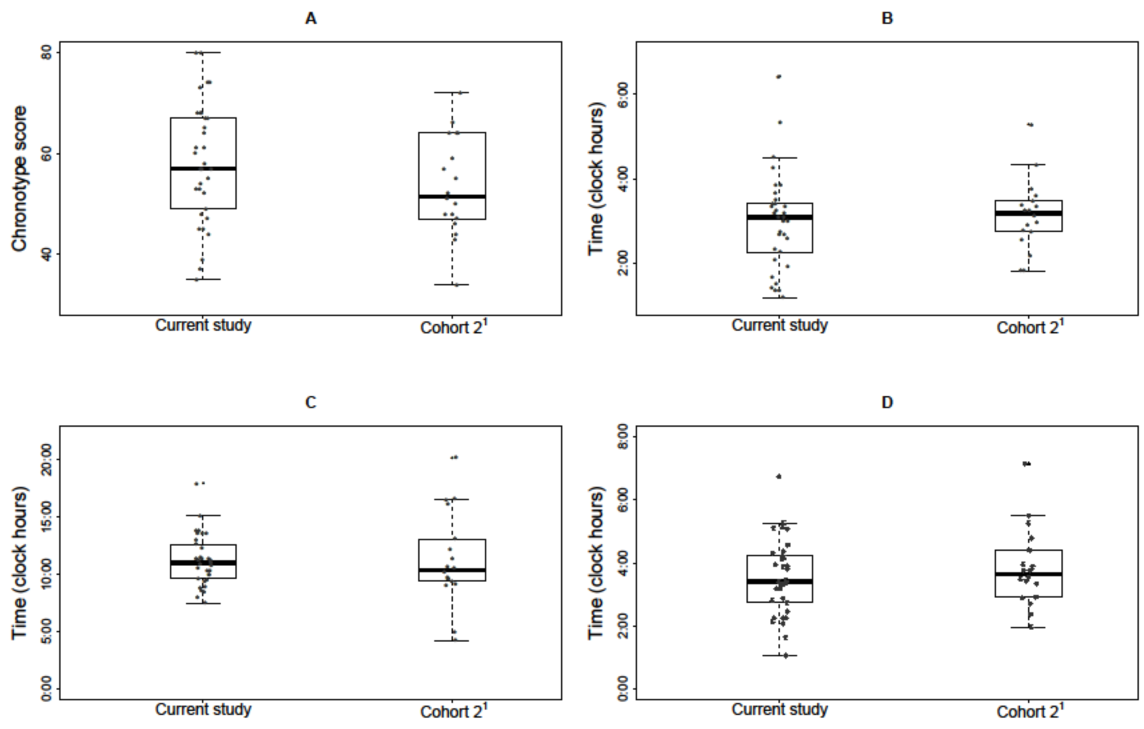


Panel A: Boxplots of chronotype scores for subjects in current study (left N=33) and cohort 2(1) (right N=18).

Panel B: Boxplots of center-of-rest times.

Panel C: Boxplots of chest surface temperature bathyphase.

Panel D: Boxplots of predicted core body temperature bathyphase using the INTime model.

## References

1. Komarzynski S, Huang Q, Innominato PF, Maurice M, Arbaud A, Beau J, et al. Relevance of a Mobile Internet Platform for Capturing Inter- and Intrasubject Variabilities in Circadian Coordination During Daily Routine: Pilot Study. *J Med Internet Res.* 2018;20(6):e204.
